# Weightlessness leads to an increase granulosa cells in the growing follicle

**DOI:** 10.1038/s41526-024-00413-4

**Published:** 2024-06-22

**Authors:** Anna Yu. Kikina, Mariia S. Matrosova, Elena Yu. Gorbacheva, Ksenia K. Gogichaeva, Konstantin A. Toniyan, Valery V. Boyarintsev, Oleg V. Kotov, Irina V. Ogneva

**Affiliations:** 1 Gagarin Research and Test Cosmonaut Training Center, 141160 Star City, Moscow Region Russia; 2 Radiology Department, European Medical Center, 129090 Moscow, Russia; 3 Research Center of Neurology, 125367 Moscow, Russia; 4 Cell Biophysics Lab, State Scientific Center of the Russian Federation Institute of Biomedical Problems of the Russian Academy of Sciences, 123007 Moscow, Russia; 5 Gynecology Department, FGBU KB1 (Volynskaya) UDP RF, 121352 Moscow, Russia; 6 Emergency and Extreme Medicine Department, Central State Medical Academy UDP RF, 121359 Moscow, Russia; 7 Medical and Biological Physics Department, I.M. Sechenov First Moscow State Medical University, 119991 Moscow, Russia

**Keywords:** Medical research, Quality of life

## Abstract

The participation of women in space programs of increasing flight duration requires research of their reproductive system from the perspective of subsequent childbearing and healthy aging. For the first time, we present hormonal and structural data on the dynamics of recovery after a 157-day space flight in a woman of reproductive age. There were no clinically significant changes in the reproductive system, but detailed analysis shows that weightlessness leads to an increase in the proportion of early antral follicles and granulosa cells in large antral follicles. Returning to Earth’s gravity reduces the number and diameter of early antral follicles.

## Introduction

The exploration of deep space will inevitably lead to an increase in the duration of space flights and the establishment of bases on other celestial bodies of the Solar System. Currently, most space explorers are of reproductive age, and despite the historical partiality towards men, recent trends indicate a growing proportion of women in space programs. However, women participating in the space programs usually delay childbearing until finishing their flight career^1^, although their average age at this point is approaching 40 years^2^. Moreover, healthy aging in women significantly depends on steroid hormones, the majority of which are synthesized in the ovaries.

The available data on the functioning of women’s reproductive system after space flight and the influence of various factors on it, primarily weightlessness, are insufficient for definite conclusions^3^. Sometimes women use oral contraceptives during space flight^4^ to facilitate comfort in a confined environment, which hinders the evaluation of follicle and endometrial growth, as well as hormonal levels. Concurrently, a documented case of jugular vein thrombosis in a female astronaut^5^ has provided a rationale for considering alternative approaches or potentially abandoning cycle suppression during spaceflight missions^6^.

Men also more frequently participate in ground-based model experiments (bed-rest and “dry” immersion), resulting in limited data on the state of women’s reproductive system. A 17-day bed-rest study revealed that the length of the menstrual cycle remains unchanged^7^, although a decrease in progesterone concentration was observed in some subjects^8^. Furthermore, a 5-day ‘dry’ immersion study indicated no change in the length of the menstrual cycle^9,10^. However, according to our data, the diameter of growing follicles and the concentration of inhibin B increase, while the level of luteinizing hormone (LH) and progesterone decrease in the early follicular phase^9^.

The predominance of ground-based experiments involving female participants are relatively short-term and merely simulations, so a real space flight remains the sole opportunity to obtain data on the cumulative impact of all its factors on the female reproductive system. Therefore, in this study we present unique data on the reproductive system of a Russian female cosmonaut (with a focus on the follicular phase), who made her first 157-day space mission.

## Results

### Study design

**Fig. 1:**
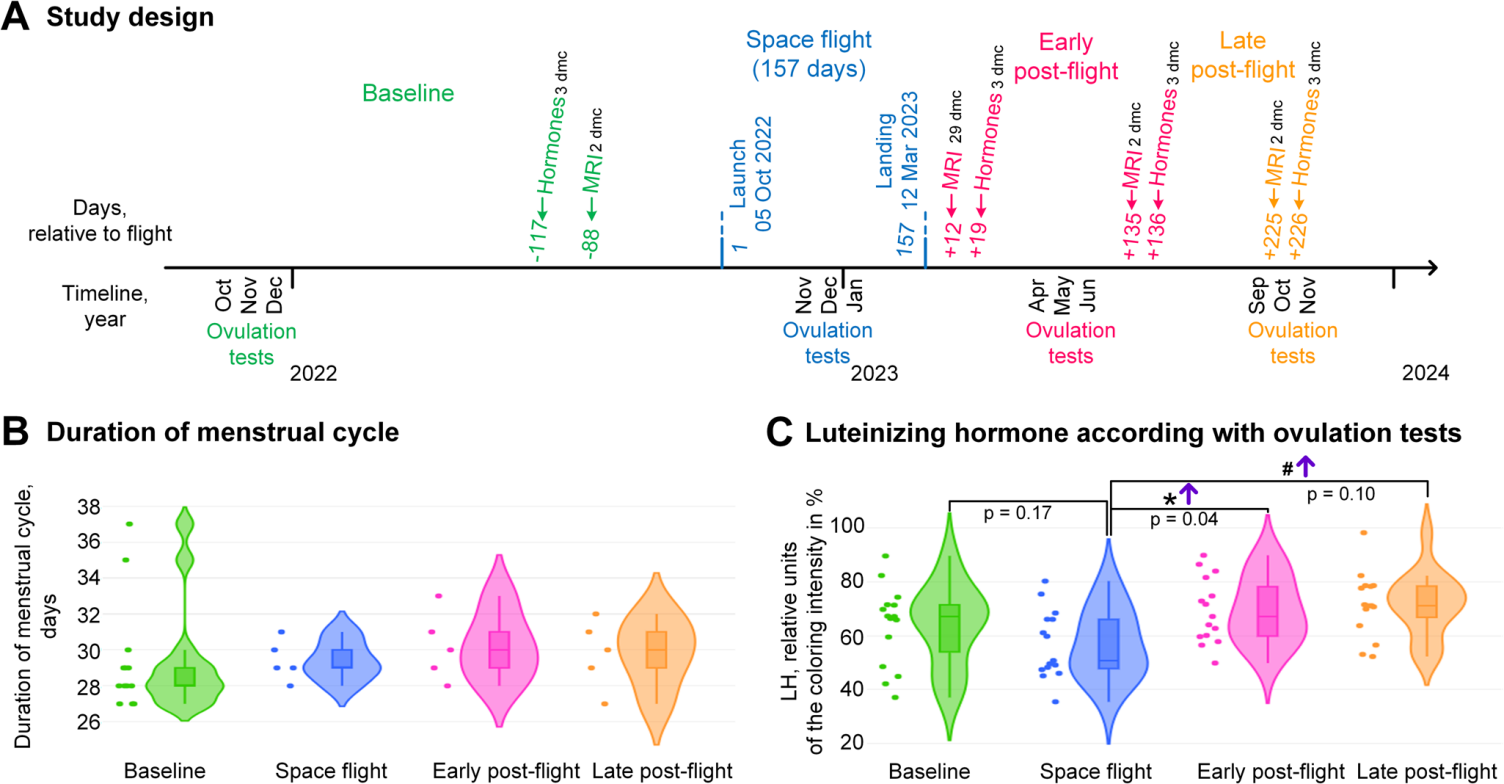
Experimental design and the menstrual cycle characteristic: its duration and luteinizing hormone at the ovulatory phase. Panel (**A**) shows time points of the data collection during the study. Baseline (green)—period before launch, space flight (blue) – from launch to landing, early post-flight (pink)—during 200 days after landing, late post-flight period (orange)—from 200 days after landing up to 325 days. Hormone concentration in blood samples was determined on the 3rd day of menstrual cycle (dmc). MRI was provided usually on the 2nd dmc, expect just after the space flight—the first MRI in the early post-flight period was on the 29 dmc. Ovulation tests were made from the 12 dmc to the 16 dmc over 3 months in each data collection period. Panel (**B**) shows the menstrual cycle duration, with median values derived from 5 to 6 cycles in each period of the data collection, for the baseline data—it was during 13 cycles. Panel (**C**) shows relative concentration of the luteinizing hormone according to ovulation tests at the ovulatory phase of menstrual cycle. * – *p* < 0.05—significant difference between the space flight period and the early post-flight period, ^#^ – *p* = 0.1—tendency between the space flight period and the late post-flight period. Due to the technical limitations and considerable variability of this method we have provided the exact meaning of the probability for comparisons as well as primary images in the supplementary materials (Supplementary Fig. 2).

Russian female cosmonaut K., aged 38, with no prior pregnancies and not using oral contraceptives, completed her first 157-day space flight. The cumulative absorbed dose was 60.24 mGy according to personal dosimetry. Baseline data collection began one year before launch (Fig. 1A). Due to the lack of facilities for blood collection and sex hormone analysis in the Russian segment of the International Space Station, ovulation tests (with sensitivity at the level of 25 mIU/ml) were used to determine the presence of ovulation and assess the relative content of luteinizing hormone in urine. These tests were carried out for 3 consecutive months from the 12th to the 16th day of the menstrual cycle before the flight, during the flight and twice post-flight. Since the follicle growth period from primordial to ovulatory spans approximately 200 days^11^, we divided the post-flight period into two phases: early and late post-flight.

Blood samples for hormone concentration analysis by immunoassays were collected on the 3rd day of menstrual cycle (dmc) in the baseline period (117 days before launch), twice in the early post-flight period (19 and 136 days after landing) and once in the late post-flight period (226 days after landing).

Pelvic magnetic resonance imaging (MRI) examinations were performed usually on the 2nd dmc before launch (–88 days) and after landing (+135 and +225 days). An additional MRI shortly after the landing (+12 days) was performed at 29 dmc (late luteal phase) due to extremely busy schedule of the cosmonaut.

For all statistically viable comparisons between the “before” and “after”, non-parametric methods were employed, with statistical significance defined at the *p* < 0.05 level and tendency—at the *p* < 0.1 level.

Written informed consent was obtained from the cosmonaut before every blood sampling and MRI. The study design and procedures were approved by Human Research Multilateral Review Board (HRMRB, protocol #21-005, signed 27 Oct 2021) and conformed to the Declaration of Helsinki.

### General characteristics

The duration of the menstrual cycle remained consistent throughout all study periods, including space flight, and averaged 29–30 days (Fig. 1B, Supplementary Fig. 1) with ovulation occurring in every cycle (Supplementary Fig. 2). During space flight, the relative peak levels of LH (in accordance with ovulation tests) could be slightly lower compared to the baseline period (*p* = 0.17, Fig. 1C), but this difference was not significant. After the space flight, in the early post-flight period, the peak relative content of LH significantly increased (*p* = 0.04) in comparison with the flight period and decreased in the late post-flight period, remaining, however, higher (*p* = 0.10) than during the flight (Fig. 1C).

According to MRI data, the structural parameters of the reproductive system’s organs did not differ before and after the space flight, staying within normal limits for the corresponding day of the menstrual cycle (Table 1).

**Table 1:** Structural parameters of the reproductive system by MRI

Parameter	Time point	Baseline -88 days (2 dmc)	Early post-flight +12 days (29 dmc x)	Early post-flight +135 days (2 dmc)	Late post-flight +225 days (2 dmc)
Uterus size, mm		62 × 33 × 41	60 × 33 × 60	55 × 28 × 40	51 × 31 × 43
Endometrium thickness, mm		4.88	8.75	4.00	5.30
Right ovary size, mm		38 × 27 × 27	35 × 18 × 19	37 × 20 × 27	38 × 22 × 23
Right ovary volume, ml		14.5	6.3	10.5	9.8
Left ovary size, mm		31 × 19 × 23	46 × 26 × 22	35 × 17 × 29	38 × 20 × 25
Left ovary volume, ml		6.8	13.8	9.0	9.9
Average ovary volume, ml		10.6	10.0	9.7	9.9
Max diameter of follicle in the right ovary, mm		7.8	6.2	8.1	7.5
Max diameter of follicle in the left ovary, mm		6.2	23 (*corpus luteum*)	7.7	9.1

In this table we provide data concerning general structural parameters of the reproductive system. MRI were performed on the 2 dmc (early follicular phase), except second MRI (early post-flight period, 12 days after landing—it was 29 dmc, late luteal phase, a *corpus luteum* was visualized). All of these time points are not optimal but it was the only opportunity to make this study due to very busy schedule of the cosmonaut before and just after the flight. But the main focus of the study was follicle growth. There a few theories about human folliculogenesis, but the “wave” theory is the most widespread and according with it, new wave starts at the luteal phase^12^. That is why we used the second MRI for our main purposes of the study.

### Hormone concentration

Prior to the space flight the level of anti-Mullerian hormone (AMH) was slightly higher than upper limit of the normal reference range (Table 2). After spaceflight, the AMH level decreased by approximately 20% and did not change further throughout the postflight period.

**Table 2:** Hormones involved in the functioning of the female reproductive system^a^

^a^The hormone levels were measured on the 3rd day of menstrual cycle. The meanings which are marked by red color were outside the range for the 3rd day of menstrual cycle.

Inhibin B level in the first cycle post-flight was 2 times higher than the baseline level, subsequently decreasing, but even in the late post-flight period remained 1.5 times higher than pre-flight level.

Follicle-stimulating hormone (FSH) levels increased by 30% in the early post-flight period, but then returned to the baseline level. But basal LH levels (3dmc) were 20% lower than pre-flight levels 19 days after the flight, and 40% lower after both 136 and 226 days.

Before the flight, the basic level of the progesterone hormone was more than two times higher than the normal value for that day of the menstrual cycle, but after the flight this level decreased by around 25%, but it was still above the normal reference values. At the same time, its metabolite, 17-OH-progesterone, which was also above the norm before the flight, increased even more after 19 days after landing, but normalized after 136 days. Androstenedione level, while remaining within normal limits, fluctuated in the post-flight period relative to the baseline data.

These fluctuations in androstenedione, a precursor hormone, did not impact the levels of total and free testosterone. Although the testosterone metabolite, 17β-estradiol, was 50% higher 19 days post-flight compared to pre-flight levels, it increased to over twice the baseline level after 136 days, but returned to the baseline after 226 days.

**Fig. 2:**
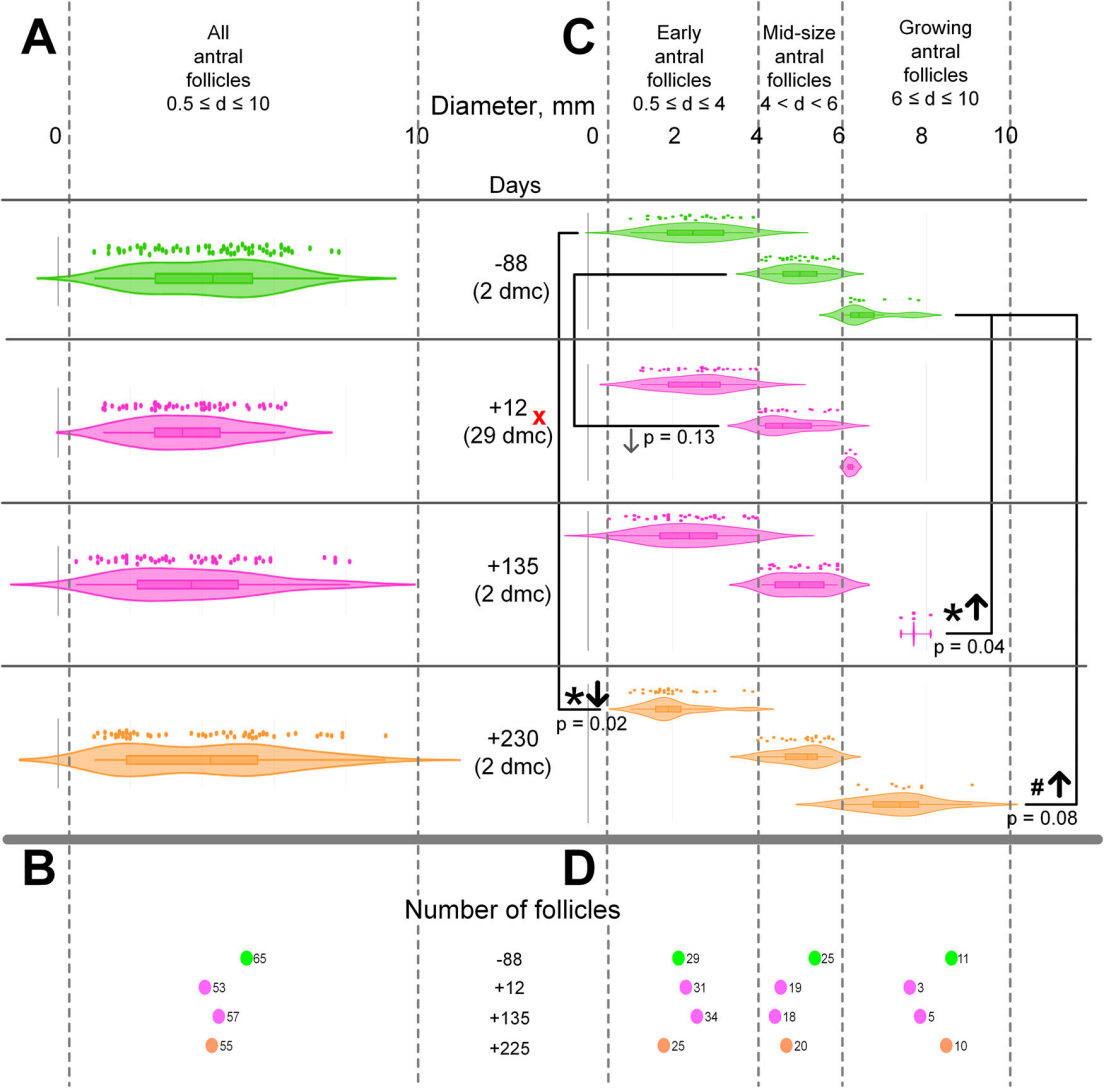
Diameter and number of the antral follicles according to MRI. Baseline data (88 days before space flight) are green, early post-flight (19 days and 135 days after space flight)—pink, late post-flight (225 days after space flight)—orange. * – *p* < 0.05 in comparison with the baseline data. # – *p* < 0.1 in comparison with baseline data. All MRI scans were made on the 2nd dmc—early follicular phase, except the early post-flight MRI (12 days after landing)—it was on the 29th dmc (marked as x) – late luteal phase. For early and mid-size antral follicles second MRI data were suitable for relevant comparison with baseline data. Growing antral follicles are recruited to the FSH-dependent growth within the current menstrual cycle. Hence, the second MRI data are not relevant due to a different phase of the menstrual cycle. To prevent any confusion, we did not provide statistical analysis for both the entire cohort of antral follicles and the sub-cohort of growing antral follicles. Panel (**A**) shows the diameters of follicles from the cohort of all antral follicles throughout the post-flight period. Panel (**B**) shows the number of measured follicles (means are near the colored circles). Panel (**C**, **D**) show diameters and number, respectively, of follicles from sub-cohorts (early, mid-size and growing), separated by gray dotted lines.

### Antral follicles: number and diameter (Fig. 2)

There were no significant changes in the average diameter of all antral follicles (Fig. 2A), however, their total number decreased by 15-20% after the space flight (Fig. 2B). To identify the contribution of different sub-cohorts, we separately analyzed the diameters (Fig. 2C) and number (Fig. 2D) of follicles within these sub-cohorts.

The average diameter and number of early antral follicles (0.5 mm ≤d ≤ 4 mm) just after the space flight and in early post-flight period matched those of the baseline period. But in the late post-flight period their number decreased by about 15% with significant (*p* = 0.02) reduction in their average diameter.

There were no significant changes in the diameter of follicles from the cohort of mid-size antral follicles (4 mm <d < 6 mm). These data were received on the 29th dmc, but as the growth of this cohort starts in the late luteal phase^12^ we can still consider the second MRI data despite the different day of menstrual cycle. However, the number of follicles in this cohort decreased by 25% in all data collection points after the space flight.

When comparing the early post-flight period (+135 days) to pre-flight (-88 days), while the total number of actively growing follicles was halved (5 compared to 11), the average diameter of actively growing follicles (with a diameter ≥6 mm) was significantly higher (*p* = 0.04). However, evaluating the late post-flight period, neither the number (10 versus 11) or diameter (*p* = 0.08) of actively growing follicles differed significantly from pre-flight values.

## Discussion

In assessing the integral state of the reproductive system of cosmonaut K. before, during, and after space flight, we conclude that while small changes are noted, that 157 days of exposure to the stressors of space, including weightlessness and galactic cosmic radiation, did not lead to clinically significant alterations in reproductive hormone levels or characteristics of antral follicles. Furthermore, our data reveals that cosmonaut K. was ovulating regularly during spaceflight and postflight period.

The growth phase from primordial to small antral follicle takes about 200 days, meaning the sub-cohort in the early post-flight period consists of follicles recruited approximately at the start of the flight – their number has not changed and even increased (Fig. 2D). The latter may be associated with a decrease in AMH (Table 2), which suppresses the recruitment of primordial follicles.

At the same time, the efficiency of transitioning to active FSH-dependent growth is reduced, since the number of mid-size and growing follicles was less than before the flight (Fig. 2D). This pattern may be associated with a decrease in the number of FSH receptors on the granulosa cells of early antral follicles, which results in decreased uptake and consequently, an increased concentration of FSH in the bloodstream, observed in this case (Table 2).

However, follicles that nevertheless enter into the active growth (sub-cohort with a diameter ≥ 6 mm) have an average diameter larger than before the flight (Fig. 2C). An increase in the content of inhibin B (Table 2) during this period indicates that the increase in diameter is associated with a higher number of granulosa cells, where estradiol synthesis occurs, leading to elevated estradiol levels in the bloodstream (Table 1).

An increase in estradiol synthesis leads to a higher utilization of its precursors. The direct precursors of estradiol are testosterone, the concentration of which does not change, and androstenedione (via estrone), the level of which decreases in the early post-flight period by nearly 40% compared to baseline data, then increases and then decreases again (Table 2). In turn, the precursors of androstenedione, progesterone and 17-OH-progesterone, exhibit a complementary pattern: initially, 17-OH-progesterone increases while progesterone decreases and subsequently the inverse occurs. In other words, based on the hormonal data, it can be assumed that the efficiency of steroidogenesis in the progesterone - 17-OH-progesterone - androstendione—17β-estradiol axis increases, leading to a wave-like accumulation of each successive product.

It is noteworthy that in the pre-flight period, the progesterone level was almost 2 times higher than the established population norm for the corresponding phase of the menstrual cycle. The progesterone level may be influenced by the phase of the athletic training cycle in professional athletes^13^, therefore we hypothesize that the observed alterations in the astronaut’s hormonal profile following crew assignment could be associated with changes in physical activity pattern, as other indicators of the reproductive function remained within normal ranges.

The progesterone is synthesized by theca cells in an LH-dependent manner. Consequently, in the early follicular phase, LH uptake increases leading to a decreased concentration in the blood decreases (Table 2). This decrease leads to an intensification of LH synthesis by the pituitary gland, which, in the ovulatory phase, may be reflected in increased relative peak concentration (Fig. 1C). It is noteworthy that we observed a similar reproductive system profile after undergoing 5-day “dry” immersion, characterized by a significant increase in inhibin B content and a decrease in progesterone and LH levels^9^.

In the late post-flight period, it is possible that not all, but some follicles greater than 6 mm in diameter are recruited during space flight–the average diameter of this sub-cohort decreases compared to the early post-flight period, yet remains slightly higher than before the flight (Figure 3C). Similarly, inhibin B also remains higher, although estradiol levels return to a baseline (Table 2).

At the same time, the number of follicles in this cohort increases and does not differ from the baseline period (Fig. 2D) against the background of a decrease in FSH also to the pre-flight value (Table 2). However, in the early antral follicles’ cohort (they were recruited to grow after landing), both the number and the average diameter are reduced (Fig. 2C, D), even though AMH level remains reduced as in the early post-flight period. This effect can hardly be associated with the cumulative absorbed dose of ionizing radiation (for cosmonaut K. it was 60.24 mGy), as the number of follicles recruited in the last quarter of the flight even exceeds the baseline level.

In summary, based on our prior data from a 5-day ‘dry’ immersion study^9^ and the findings of this research, we have accrued further evidence to hypothesize that weightlessness leads to an increase in the recruitment of primordial follicles and an increase in the number of granulosa cells in follicles that have entered the intensive growth stage. Nonetheless, we anticipate the acquisition of a larger statistics and more robust measurements of hormone levels across various stages of the menstrual cycle to reinforce these observations. On the one hand, this is a good prognostic indicator of the follicular phase viability, but, on the other hand, with prolonged exposure it may accelerate the depletion of the ovarian reserve. Returning to Earth’s gravity reduces recruitment efficiency and slows granulosa cell division. Both processes are regulated by the growing oocyte, which produces TGFβ family factors, GDF9 (apparently does not change in microgravity, at least in mice^14,15^) and BMP15, along with other paracrine factors^16^. Nevertheless, further research is essential to fully elucidate the underlying mechanisms.

## Methods

### Magnetic resonance imaging (MRI)

All pelvic MRI investigations were performed without any special preparing at the 2nd dmc with exception of the MRI performed 19 days after flight (day 29; late luteal phase) given the cosmonaut’s extremely inundated schedule. All images were received by the MRI scanner Siemens Magnetom (3 T), using standard sequences: T1, T2, T2 stir, diffusion-weighted imaging (DWI) with apparent diffusion coefficient (ADC) mapping in axial, coronal and sagittal planes with the slice thickness of 4 mm. To determine structural parameters of the reproductive system (the size of the uterus and ovaries, thickness of endometrium, the diameter of the follicles and counting their number) all images were proceeded using the integrated DICOM viewer SyngoVia.

### Blood sampling and measurements of hormones concentration

Blood samples were collected around 8.30 am on an empty stomach at the 3rd dmc without power training the day before. Serum was separated by centrifugation and measurements were made immediately without freezing in commercially available certificated clinical laboratory.

Follicle-stimulating hormone (FSH), luteinizing hormone (LH), progesterone (PG), 17-hydroxyprogesterone (17-OH-progesterone), androstendione, testosterone free and full, estradiol (17β-estradiol, E2), globulin, connecting sex hormones, anti-Mullerian hormone (AMH) were determined by chemiluminescence immunoassay. Dehydroepiandrosterone sulfate (DHEA-S) and inhibin B were measured by immunoenzyme assay at the automatic analyzer.

### Ovulation tests

There were no opportunity for blood sampling and measurements of sex hormones concentration on the board of Russian Segment of International Space Station. That is why we decided to use ovulation tests for estimation relative content of the luteinizing hormone in urine at least. The commercially available tests with sensitivity at the level of 25 mIU/ml were used during baseline period, space flight, early- and late-post flight periods 3 months in a row from the 12 dmc to the 16 dmc (ovulation phase). The image of test was analyzed by Fiji software. The color intensity of target band and reference band were measured, then their ratio was performed in the percent.

### Statistical analysis

We used non-parametric statistics to compare “before” and “after”. For repeated measurements of the luteinizing hormone relative content according with ovulation tests Wilcoxon signed-rank test was used. To compare follicle diameter between groups of data we used Kruskal-Wallis test. In both cases statistically significance was postulated at the *p* < 0.05 level and tendency – at the *p* < 0.1 level. Data were presented as median ± range.

### Supplementary information

Supplemental material

## Data Availability

All data generated or analyzed during this study are included in this article and Supplementary Materials. All relevant data are available from the authors.
