# Supplementary material for: Weightlessness leads to an increase granulosa cells in the growing follicle
Source: NPJ Microgravity. 2024 Jun 22;10:70. doi: 10.1038/s41526-024-00413-4 (PMC11193763; doi:10.1038/s41526-024-00413-4)

Supplementary Figure 1. Detailed study design in the connection with menstrual cycle. Here we provide the full timeline of the study with exact dates of the blood collection for hormone estimation, MRI and ovulation tests in the connection with days of menstrual cycle. On the histogram columns with more intensive color mean the months where ovulation tests were made.

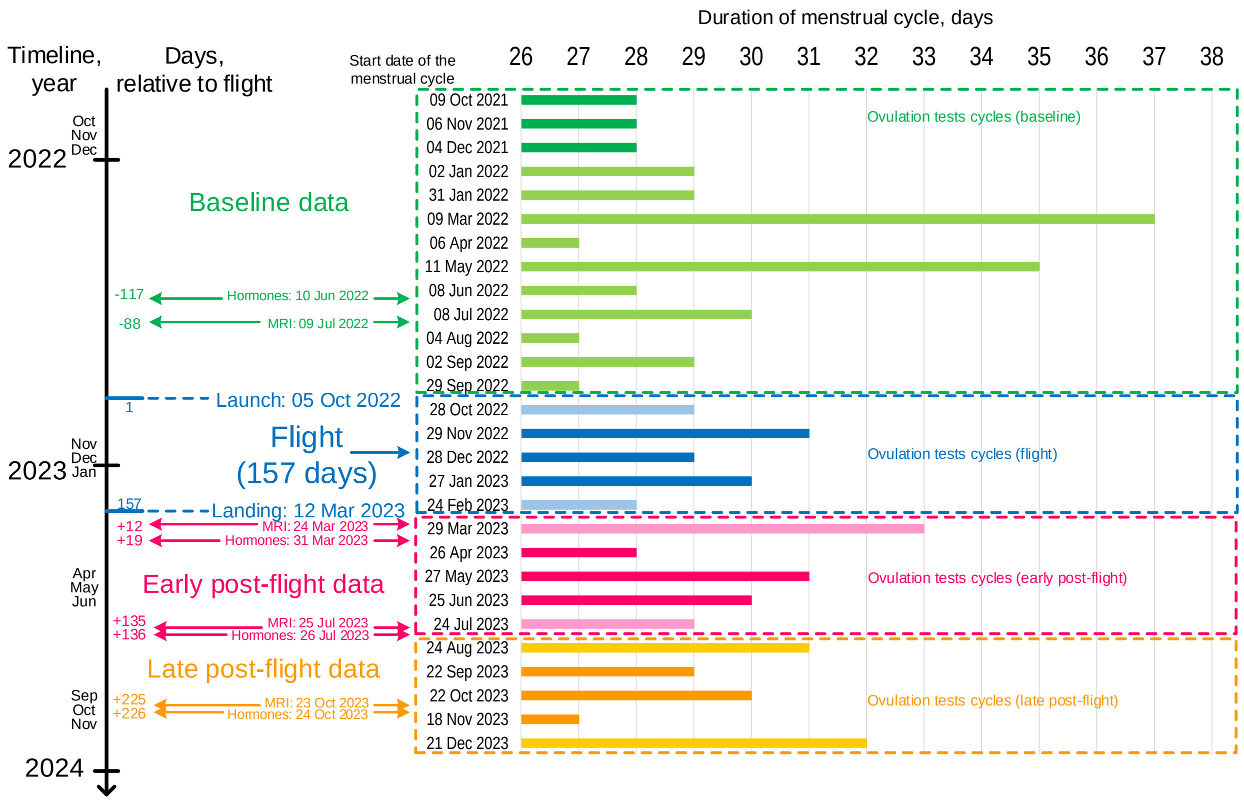

Supplementary Figure 2. Ovulation tests data.

Here we provide primary images of the ovulation tests which were made from the 12nd day of menstrual cycle (dmc) to the 16th dmc with exact dates during baseline data collection, space flight, early and late post-flight periods. To estimate luteinizing hormone relative content, we used semi-quantitive method: left band on the test is target, right – reference. We estimated intensity of coloring each band and normalize for each test left band to right band and express this evaluation in percent. These data were used for building graphics presented under the tests. During each period of data collection three month in a row, tests were made and for every month graphic is presented.

Baseline

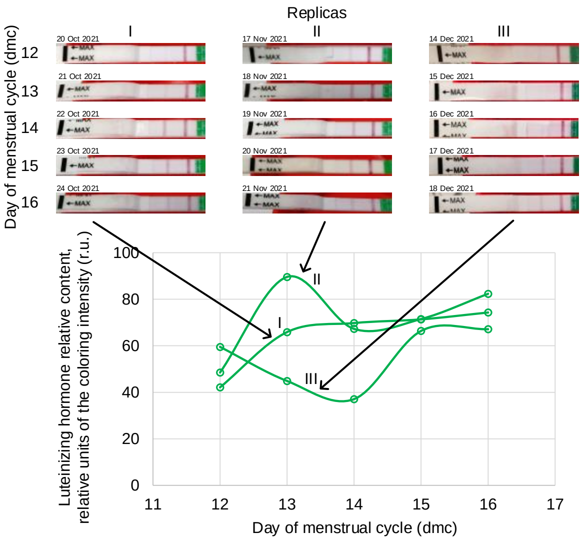

Flight

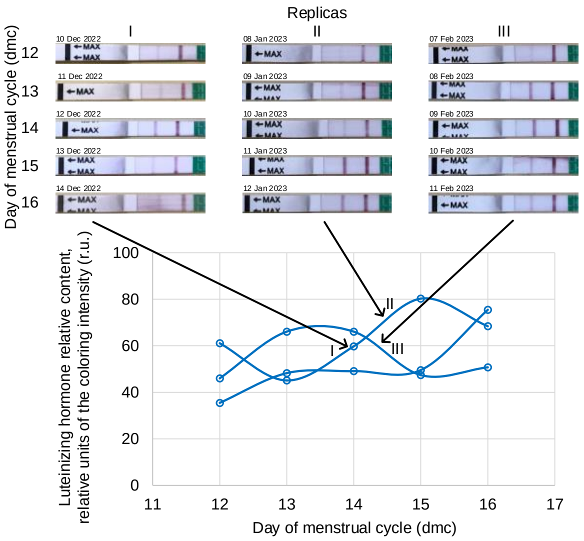

Early post-flight

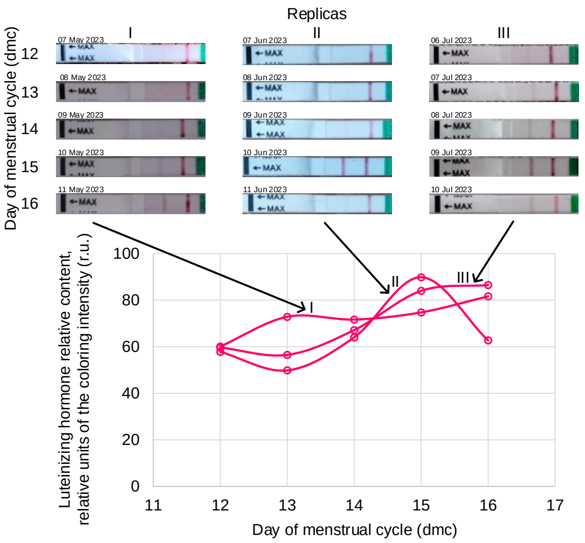

Late post-flight

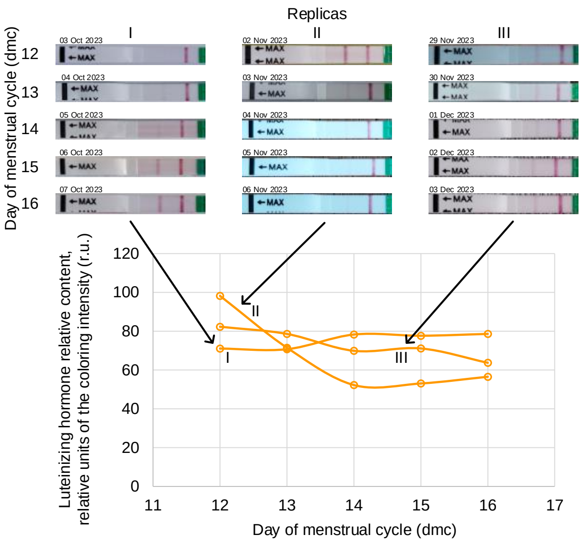

Supplement: Supplementary file 1 — Supplemental material [file 41526_2024_413_MOESM1_ESM.pdf]
